# Potential Roles of Gamma-Delta T Cells in a Bacterial Immun-Ization Model

**DOI:** 10.3390/vaccines14070590

**Published:** 2026-07-01

**Authors:** Lee Anne Talbot, Raffi Manjikian, Constantine Bitsaktsis

**Affiliations:** 1 Department of Biological Sciences, Seton Hall University, South Orange, NJ 07079, USA; leeanne.talbot@student.shu.edu; 2 Instructor of Chemistry, School of STEM, Hudson County Community College, Jersey City, NJ 07306, USA; rmanjikian@hccc.edu

**Keywords:** gamma-delta (γδ) T cells, mucosal immunity, intranasal immunization, pulmonary immunity, lung-resident memory cell formation

## Abstract

**Background/Objective:** Francisella tularensis is a highly infectious intracellular pathogen that causes severe pulmonary tularemia following aerosol exposure, yet no licensed vaccine exists. Because infection initiates at the respiratory mucosa, understanding mechanisms of protective pulmonary immunity is critical for mucosal vaccine development. This study investigated the role of lung-resident γδ T cells following intranasal immunization with inactivated *F. tularensis* (i*Ft*) and subsequent lethal challenge with live vaccine strain (LVS). **Methods:** Mice were intranasally immunized with i*Ft* and later challenged with lethal LVS. Pulmonary immune responses were evaluated using flow cytometry and cytokine analysis. Recruitment of γδ and αβ T cells, production of IL-17 and IFN-γ, neutrophil infiltration, and γδ T cell memory phenotypes were assessed in naïve and immunized mice following infection. **Results:** Primary LVS infection induced rapid recruitment of γδ T cells to the lung beginning on Day 2 post-infection, preceding significant αβ T cell accumulation. Increased pulmonary IL-17 and IFN-γ correlated with expansion of IL-17– and IFN-γ–associated γδ T cell populations. Following i*Ft* immunization, mice demonstrated enhanced survival after lethal LVS challenge, accompanied by early increases in pulmonary IL-17 and IL-17 producing γδ T cells. Immunized mice also exhibited expansion of effector memory and central memory γδ T cell populations associated with IL-17 production. **Conclusions:** These findings identify IL-17 producing γδ T cells as contributors to early mucosal immunity following intranasal vaccination against *F. tularensis* and suggest that targeting lung-resident γδ T cells may support the development of next-generation mucosal vaccines against respiratory pathogens.

## 1. Introduction

*Francisella tularensis* (*F. tularensis*) is a highly infectious intracellular Gram-negative bacterium and the causative agent of tularemia. Pulmonary tularemia, which results from inhalation of aerosolized organisms, represents the most severe clinical manifestation and is associated with substantial mortality if untreated [1]. In natural settings, transmission occurs through contact with infected wildlife or inhalation of contaminated aerosols [1]. Disease severity varies depending on the route of infection, with pulmonary and typhoidal forms exhibiting severe toxicity. Due to its low infectious dose, efficient airborne transmission, and potential for large-scale dissemination, *F. tularensis* has long been recognized as a significant biodefense concern [2]. Despite this, no licensed vaccine currently exists.

A major obstacle to vaccine development is the capacity of *F. tularensis* to evade early host immune responses. During the initial stages of infection, the bacterium suppresses inflammatory signaling and promotes anti-inflammatory cytokine production, thereby delaying activation of innate and adaptive immunity [3,4]. Under physiological conditions, pattern recognition receptors (PRRs) detect conserved microbial structures and initiate pro-inflammatory cytokine and chemokine responses that coordinate host defense [5]. However, *F. tularensis* either fails to robustly activate PRRs or engages pathways that limit inflammation, thereby enabling intracellular replication [5]. Consequently, early immune events at mucosal surfaces are likely critical determinants of disease outcome and protective immunity.

The live attenuated vaccine strain (LVS) of *F. tularensis* remains the most widely used experimental model for studying tularemia. LVS reproduces key aspects of human disease and is lethal in mice, allowing mechanistic investigation of pulmonary infection under biosafety level 2 conditions. This model provides a platform to define immune correlates of protection and to evaluate mucosal vaccination strategies.

Recent work has highlighted the importance of immune populations capable of rapid effector function in barrier tissues. γδ T cells represent a distinct T lymphocyte subset that bridges innate and adaptive immunity [6]. Unlike conventional αβ T cells, γδ T cells recognize antigen independently of major histocompatibility complex (MHC) restriction and respond rapidly without prior clonal expansion [7,8]. Although they constitute a small fraction of circulating T cells, γδ T cells are enriched in epithelial and mucosal compartments, including the lung, where they contribute to tissue homeostasis and early immune responses [9,10]. Functionally, γδ T cells modulate both naïve and memory αβ T cell responses, help B cells, act as antigen-presenting cells, and produce cytokines associated with Th1, Th2, and Th17 profiles [11,12,13]. Their strategic localization and rapid cytokine production suggest that they may play a pivotal role in shaping pulmonary immunity during infection.

Clinical and experimental studies support this concept. Increased frequencies of circulating γδ T cells have been reported during acute tularemia, with persistence following recovery [14,15]. Similarly, murine models of respiratory bacterial infection demonstrate rapid recruitment of γδ T cells to the lungs, where they contribute to early pathogen control and inflammatory activation [16,17]. Mice lacking γδ T cells exhibit increased bacterial burden, impaired neutrophil recruitment, and reduced survival, highlighting a non-redundant role in host defense. Similar γδ T cell responses have been observed across various immunization models, including BCG-vaccinated non-human primates infected with *Mycobacterium tuberculosis*, cattle infected with *Leptospira borgpetersenii*, humans exposed to *Mycobacterium* BCG, mice infected with *Staphylococcus*, and chickens challenged with *Salmonella enteritidis*, where γδ T cell activity increased upon reinfection [18,19,20,21,22]. These findings suggest that γδ T cells act as early sentinels during pulmonary infection.

Mechanistically, γδ T cell mediated protection is closely linked to cytokine production. These cells are potent sources of interferon-γ (IFN-γ) and interleukin-17 (IL-17), both of which are essential for macrophage activation, neutrophil recruitment, and bacterial containment [23]. In respiratory tularemia, early survival is dependent on innate immune pathways, including IFN-γ and neutrophil-mediated responses, rather than classical adaptive immunity [24,25]. CD4−CD8− T cells consistent with a γδ phenotype have been identified as dominant early producers of these cytokines in the lung [26,27]. Importantly, IL-17 signaling is required for neutrophil recruitment and effective bacterial clearance, underscoring the importance of this pathway in early defense [27,28,29,30,31,32,33].

Emerging evidence further indicates that γδ T cells exhibit adaptive-like features. Memory-associated phenotypes defined by CD44 and CD62L expression have been described, and γδ T cells expand upon secondary exposure to bacterial pathogens [18,19,20,21,22,34,35,36,37,38]. These observations suggest that γδ T cells may contribute to both early innate responses and adaptive mucosal immunity.

Mucosal vaccination strategies against *F. tularensis* support this idea. Intranasal immunization with inactivated bacteria (i*Ft*) induces partial protection and promotes IFN-γ production and inflammatory cell recruitment in the lung [39,40]. However, the cellular mechanisms underlying this protection remain incompletely defined. In particular, previous work identified a CD4− non-CD8+ T cell population producing IFN-γ following immunization and challenge, suggesting a potential role for γδ T cells in recall responses [4].

Based on these observations, we hypothesized that lung-resident γδ T cells contribute to early and memory-like immune responses following mucosal immunization against *F. tularensis*. The present study therefore investigated γδ T cell activation, cytokine production, and memory phenotypes following intranasal immunization with inactivated *F. tularensis* and subsequent LVS challenge. Defining the contribution of these cells to pulmonary immunity will provide insight into mechanisms of protection and may inform the rational design of next-generation vaccines targeting mucosal defense against intracellular pathogens.

## 2. Materials and Methods

### 2.1. Murine Model

C57BL/6 mice were purchased from Taconic Laboratories (Hudson, NY, USA). All mice were housed at Seton Hall University (South Orange, NJ, USA) in the Animal Research Facility (McNulty Hall). The female mice were used at 6–10 weeks of age. Mice were housed and cared for according to IACUC protocol CB1401, starting date 7 February 2019.

### 2.2. F. Tularensis Bacteria

The live vaccine strain of *F. tularensis* (LVS) was provided by Dr. Edmund Gosselin (Albany Medical College, Albany, NY). The organisms grew to a concentration of 2.6 × 10^9^ CFU/mL at 37 °C to a concentration of 2.6 × 10^9^ CFU/mL, using Mueller-Hinton broth supplemented with 2% IsovitaleX^TM^ (Fisher Scientific, Waltham, MA, USA). Live *F. tularensis* samples were stored at −80 °C in liquid nitrogen until needed.

### 2.3. Inactivation of Bacteria

Inactivated *F. tularensis* LVS (i*Ft*) was generated by growing *F. tularensis* LVS in Mueller Hinton broth (MHB) media (BD Biosciences, San Jose, CA, USA) up to a density of 1 × 10^9^ CFU/mL. The culture was then centrifuged at 22,000× *g* for 20 min at 4 °C, washed 3 times with phosphate-buffered saline (PBS), resuspended in 2% Paraformaldehyde (Sigma, Seoul, Republic of Korea) and incubated on a rocker for 2 h at room temperature. Bacteria were then washed three more times with PBS. In total, 1 × 10^9^ organisms were placed on a chocolate agar plate (BD Biosciences) at 37 °C and incubated for 7 days to confirm inactivation. The final concentration of i*Ft* organisms was determined by OD at 610 nm.

### 2.4. Dose Titration: Inflammation Study

**Figure 1:**
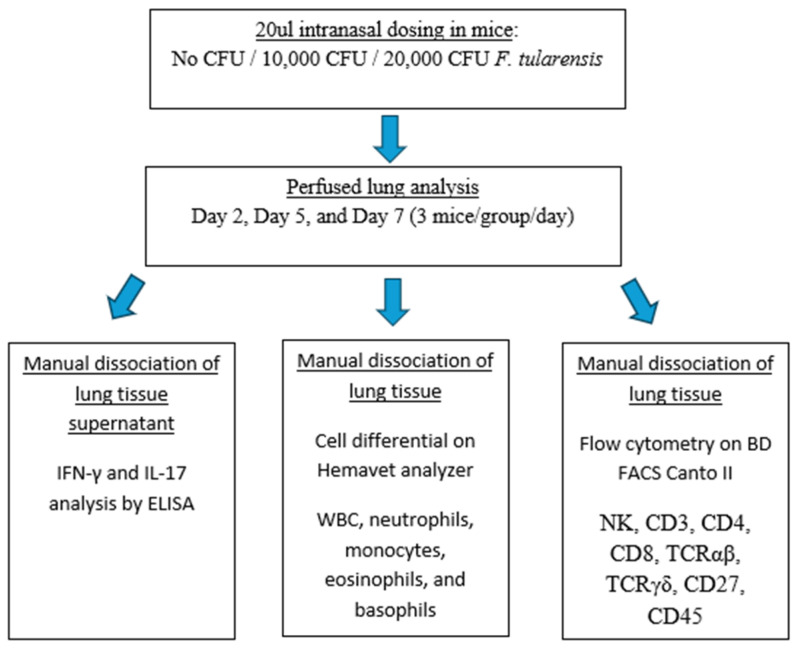
Dose Titration Procedures: Inflammation Study-The dose titration/inflammation study consisted of three groups of animals (one control and two test groups), days of perfused lung analysis, and cytokine testing, cell differentials, and flow cytometry testing on lung tissue.

Initial studies were conducted to understand the in vivo response of γδ T cells to *F. tularensis* LVS through intranasal dosing in mice and monitored for cell changes and cytokine production. In the first set of experiments, naïve C57/BL6 mice were intranasally dosed with 20 µL of 10,000 colony-forming units (CFUs) or 20,000 CFUs of *F. tularensis* LVS, the control animals were kept naïve. On days 2, 5, or 7 post-dosing, three mice from each treatment group were euthanized, and the lungs were collected. The PBS group served as a single baseline control. Figure 1 demonstrates the procedures used for dose titration.

### 2.5. Immunization

To investigate the role of γδ T cells in this immunization, studies were conducted on naïve C57BL/6 mice, following established protocols [3,4,40]. Mice were treated with either PBS (control) or inactivated *F. tularensis* LVS (i*Ft*) on Day 0, followed by a booster dose of i*Ft* on Day 21. On Day 34, thirteen days post-booster, all groups received a lethal challenge dose of 10,000 CFU *F. tularensis* LVS. Our lab has previously observed clinical symptoms in non-immunized mice seven days post-infection with this concentration, and we have confirmed activation of γδ T cells at this dose.

**Figure 2:**
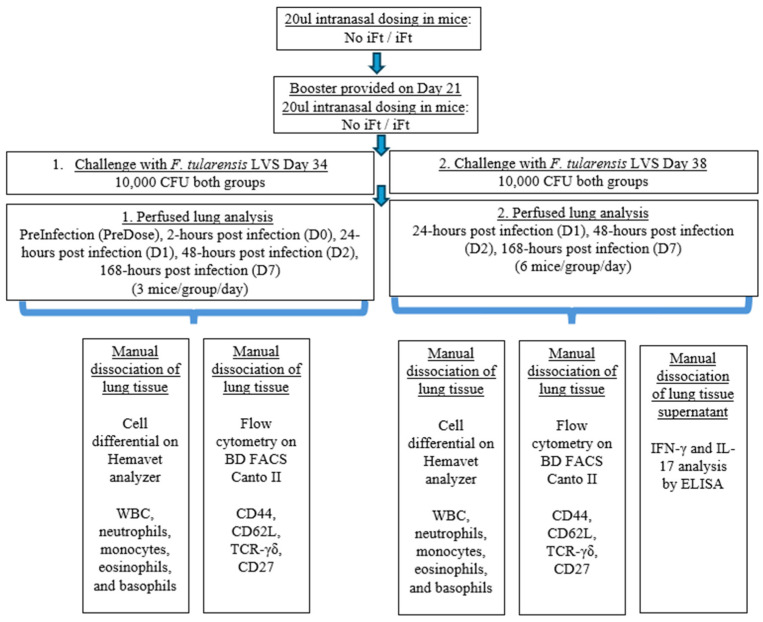
Immunization Studies Procedures: Immunization studies consisted of two groups (one control and one test group), an immunization booster for the test group, a *F. tularensis* LVS challenge in both groups, sample collection, and lung tissue analysis through cell differential, flow cytometry, and cytokine analysis.

C57BL/6 mice were divided into three groups consisting of 15 animals per group, 6–10 weeks of age. The mice were intranasally dosed on Day 0 and again on Day 21 with 20 μL of 2 × 10^7^ i*Ft*. The control group was not immunized and considered naïve. On Day 34 (Day 0 of challenge), the mice in all groups were challenged intranasally with 20 μL containing 10,000 CFU of live *F. tularensis* LVS. Three mice from each group were euthanized and the lungs collected: prior to the challenge, 2 h (Day 0), 24 h (Day 1), 48 h (Day 2), and seven days (Day 7) post-challenge with *F. tularensis* LVS. The chosen time points were informed by previous research within the Bitsaktsis lab [3,4,40]. The 2-h post-challenge collection was based on Ojeda [41], which demonstrated the detection of radiolabeled *F. tularensis novicida* in lung and digestive tissues as early as 15 min post-infection, with increased detection through the 2-h mark [41]. Figure 2 demonstrates the procedures used for immunization studies.

### 2.6. Flow Cytometry Screening

Lungs were collected and perfused with 0.02% EDTA (Teknova, Hollister, CA, USA) in DPBS (Corning, Corning, NY, USA). The perfusion was discarded. The sample was then shipped on ice packs for same-day processing and analysis to Novartis Pharmaceuticals, East Hanover, NJ. Lung tissue was processed by manual homogenization (syringe plunger and Petri dish) and pushed through a 70 µm filter. The single-celled suspension was transferred to a 5 mL polystyrene 12 × 75 tube and centrifuged at 200× *g* for 5 min at 4 °C. The supernatant was saved for future cytokine analysis, and the cell pellet was then reconstituted in 1 mL red blood cell lysis buffer (house-made: Ammonium chloride, Potassium phosphate, EDTA) for approximately two minutes (until lysis occurs) and then centrifuged at 200× *g* for 5 min at 4 °C. The supernatant was discarded, and the cell pellet was adjusted to 1 × 10^6^ cells for flow cytometer analysis.

Analysis of the washed cell pellet was conducted using a Drew Scientific Hemavet 950 analyzer which was validated for mouse blood and tissue. From the washed cell pellet, 30 µL of the concentrated lung cells was analyzed for the initial concentration of white blood cells. The pellet was then adjusted with DPBS (Corning) to a final concentration of 1 × 10^6^ of white blood cells prior to complete cell differentiation and FACS analysis.

Lung cells for cell surface marker staining were added to a titrated antibody cocktail or corresponding isotype control and incubated on wet ice, protected from light, for at least 30 min. Two milliliters of DPBS were added to each tube and centrifuge at 400× *g* for 5 min at 4 °C. The supernatant was decanted, and 0.2 mL of 2% formaldehyde was added to each tube. Tubes were gently mixed and incubated on wet ice or refrigerated, protected from light, for at least 30 min. A singlet gate was established on the FACS Canto II flow cytometer, and 10,000 events were collected. The following antibodies were purchased from BD Biosciences (San Jose, California) for FACS analysis: CD3-FITC (145-2C11 or 17A2), CD4-APC (RM4-5), CD8a-APC-Cy7 (53-6.7), NK1.1-PE (PK136), TCR αβ-Pacific Blue (H57.597), TCR γδ-PE/Cy7 (GL3), CD27-PerCP/Cy5.5 (LG.3A10), and CD45-BV510 (30-F11).

Immunophenotyping via fluorescence activated cell sorting (FACS) analysis of key cell types, including NK cells, T cells (CD3, CD4, CD8, TCRαβ, TCRγδ), and leukocytes (CD45) was performed. Further investigation of γδ T cells through FACS analysis included examination of CD27 expression, a marker known to differentiate cytokine profiles within γδ T cells. CD27+ γδ T cells typically produce IFN-γ, while CD27− γδ T cells are more likely to produce IL-17, as shown in Ribot [38]. For flow cytometry, γδ T cells producing IFN-γ will be identified as CD27+TCR γδ+, and those producing IL-17 will be CD27-TCR γδ+. The cytokine profile will aid in further understanding the γδ-T cell response due to infection from *F. tularensis* LVS.

Additional FACS analysis of CD44 and CD62L was conducted in this immunization experiment for the detection of memory T cells. To analyze the roles of central memory (CM) and effector memory (EM) γδ T cells, we will distinguish CD44+CD62L+ cells as CM T cells andCD44+CD62L− cells as EM T cells. Previous studies have indicated that CD44 expression denotes activation, while CD62L is a marker of central memory phenotype [34,38] (Figure S1).

Our analysis focuses on the γδ T cell inflammatory response. In gating CD44 and CD62L for each memory cell type, CD27+γδ T+ cells for IFN-γ production and CD27−γδ T+ cells for IL-17 production were examined, identifying memory responses among γδ T cells. Ribot [38] found that CD27− γδ T cells signaling IL-17 were often CD44^hi^CD62L^lo^ (EM), highlighting specific γδ T cell phenotypes associated with cytokine production. In line with this, Misiak [34] noted that CD27−CD44+ γδ T cells exhibited memory responses in a *Bordetella pertussis* infection model, with a significant increase in CD44+CD62L+CD27− cells post-rechallenge, a phenotype indicative of effector memory function.

### 2.7. Enzyme Linked Immunosorbent Assays (ELISAs)

Cytokine analysis was completed using BioLegend ELISA MAX™ (BioLegend, Inc., San Diego, CA, USA) for Mouse IFN-γ (Cat# 430804) and Mouse IL-17 (Cat# 432504), using a Molecular Devices SpectaMAX M5 (Molecular Devices, LLC, San Jose, CA, USA) spectrophotometer for the 96-well plate read and SoftMax Pro GxP 5.4.4 software for analysis of the concentration (pg/mL) of cytokines in the initial supernatant capture.

### 2.8. Statistical Analysis

Statistical differences among groups were analyzed using multiple *t* tests. GraphPad Prism 10 software was used for the statistical analysis (San Diego, CA, USA).

### 2.9. Ethics Statement

All animals were handled in accordance with good animal practice defined by relevant national and/or local animal welfare bodies. Briefly, mouse euthanasia was performed via CO_2_ administration followed by cervical dislocation.

## 3. Results

### 3.1. Increased Gamma-Delta T Cells Recruitment and Production of Il-17 and Ifn-Γ, in the Lungs of Mice During Primary Lvs Infection

**Figure 3:**
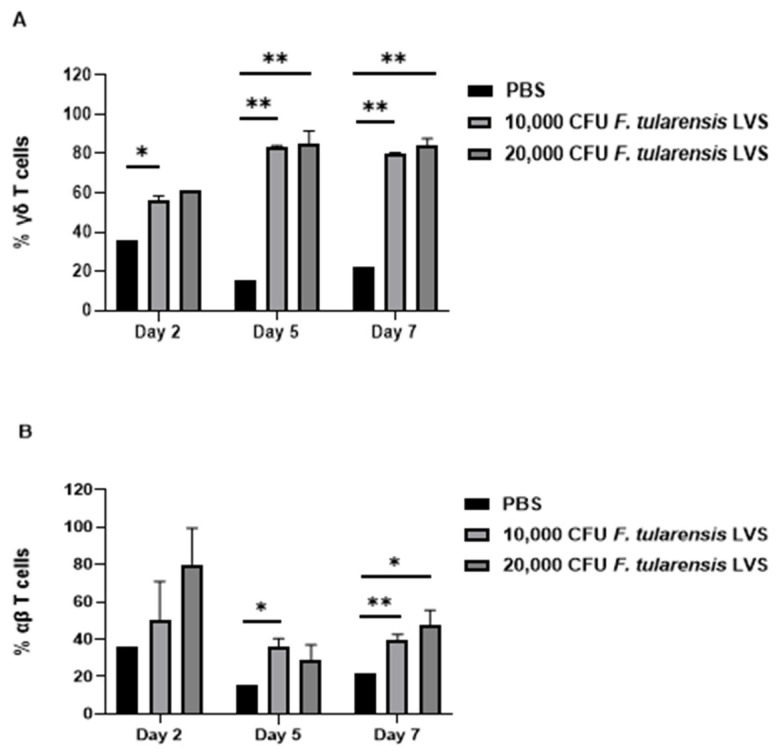
γδ and aß T cells change due to infection. Infection of the mice with either 10,000 or 20,000 CFU *F. tularensis* LVS resulted in an increase in both γδ and aß T cells. A significant increase in γδ T cells was seen by Day 2 and continued through the close of the study on Day 7 (**A**). A significant increase was seen in aß T cells on Day 5 and continued through the close of the study on Day 7 (**B**). Lungs were harvested each day, and WBC were analyzed for antibodies for TCR γδ (GL3) or TCR aß (H57.597) through FACS. Due to the variance in the absolute number with sample degradation, the results are presented as a percentage of the parent population (WBC). Three mice were used from each treatment group, each time point; the results are from one of the three experiments. (*) *p* < 0.1; (**) *p* < 0.05; bars represent Standard Deviation (SD).

Pulmonary γδ T cell responses were assessed following intranasal infection of mice with LVS. Intranasal dosing of 10,000 or 20,000 CFU of LVS significantly increased the percentage of γδ T cells (Figure 3A) by Day 2 and αβ T cells by Day 5 (Figure 3B), in the mouse lung, compared to PBS control mice. As seen in Figure 3, γδ and αβ T cells were both significantly elevated due to infection. Gamma-delta T cells, having tissue-resident properties within the lung, as well as the ability to recognize antigen independent of MHC restriction, increased significantly by Day 2 and continued to increase by Day 5 post dose, remaining elevated through the end of the study on Day 7 (Figure 3A). The αβ T cells increased with similar kinetics to the γδ T cells, albeit at a slower rate, likely due to their need for MHC antigen presentation and subsequent clonal expansion (Figure 3B).

**Figure 4:**
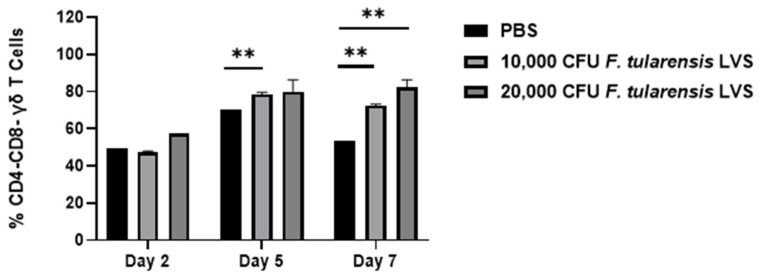
γδ T cells as a subpopulation of CD4−CD8− and the changes due to infection. Intranasal infection of mice with either 10,000 or 20,000 CFU *F. tularensis* LVS resulted in a significant increase in CD4−CD8−γδ T cells by Day 5 and continuing through the end of study on Day 7. Lungs were harvested each day, and WBC were analyzed for antibodies for CD4 (RM4-5), CD8a (536.7), and TCR Υδ (GL3) through FACS analysis. Due to the variance in the absolute number with sample degradation, the results are presented as a percentage of the parent population (CD4−CD8−). Three mice were used for each treatment group at each time point; the results are from one of the three experiments. (**) *p* < 0.05; bars represent SD.

Phenotypic analysis of γδ T cells in the lung revealed that the majority of responding cells were CD3+CD4−CD8−. This population significantly increased between Day 5 and Day 7 post-infection compared to PBS controls (Figure 4), confirming the identity of the γδ T cell population assessed and validating the gating strategy used for downstream analyses.

**Figure 5:**
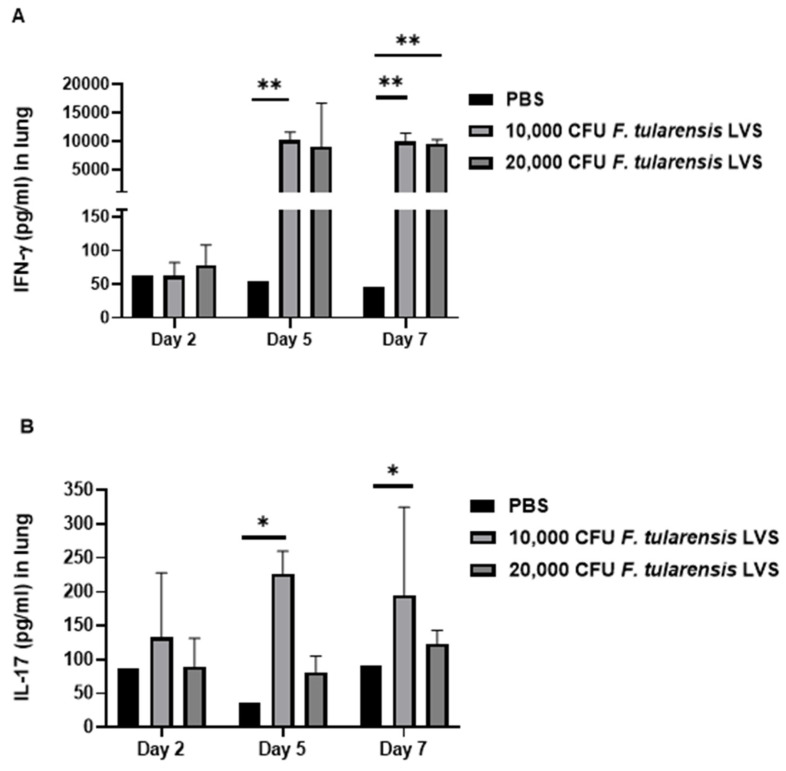
Changes in cytokine response due to infection. Infection of the mice with either 10,000 or 20,000 CFU *F. tularensis* LVS resulted in an increase in IFN-γ (**A**) and IL-17 (**B**) beginning on Day 5 and continuing through the close of the study on Day 7. Lungs were harvested each day and the supernatant from the first wash of disassociated lung cells was analyzed by ELISA for IFN-γ or IL-17 concentrations. Note: 20,000 CFU was determined to be too high of a dose for mice, causing the immune system to proceed in shutting down thus a possible reason for the decrease seen in the 20,000 CFU dosage when assaying IL-17. Three mice were used from each treatment group on each day; the results are from one of the three experiments. (*) *p* < 0.1; (**) *p* < 0.05; bars represent SD.

**Figure 6:**
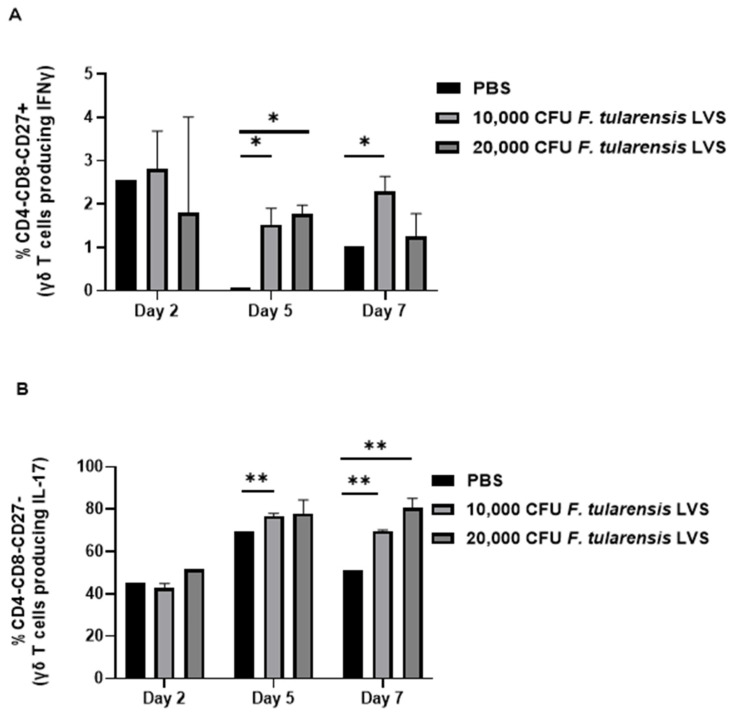
Changes in γδ T cells producing IFN-γ or IL-17 due to infection. Intranasal dosing in mice with either 10,000 or 20,000 CFU *F. tularensis* LVS resulted in a significant increase in the percentage of IFN-γ or IL-17 signaling through γδ T cells beginning on Day 5 through the end of study on Day 7 (**A**). Lungs were harvested on each day, perfused, and the cells manually dissociated and filtered. Lung cells were stained with CD4 (RM4-5), CD8a (53-6.7), and CD27 (LG.3A10). Those cells that were CD4−CD8−CD27+ are considered γδ T cells producing IFN-γ. Cells that were CD4−CD8−CD27− are considered γδ T cells producing IL-17. The percentage presented is a percentage of CD45+ (white blood cell) as the parent population (**B**). Three mice were used from each treatment group on each day; the results are from one of the three experiments. (*) *p* < 0.1; (**) *p* < 0.05; bars represent SD.

From the literature, we understand that γδ T cells predominantly produce IFN-γ and IL-17. As expected, IFN-γ and IL-17 cytokines were significantly elevated in infected mice beginning on Day 5. The significant increase in IFN-γ in the lung (Figure 5A) was correlated to an increase in CD4−CD8−CD27+ cells (possible IFN-γ producing γδ T cells, Figure 6A), which also commenced on Day 5 through the end of the study on Day 7. Similar observations were made with total IL-17, which increased in the lung tissue (Figure 5B), correlating to a significant increase in possible IL-17 producing γδ T cells (CD4−CD8−CD27−), (Figure 6B). These cytokines have been shown to be crucial in the control of respiratory bacterial pathogens [23,24,25,26,27,29].

Thus far, we have demonstrated an inflammatory response during LVS infection through recruitment of both αβ T cells and γδ T cells, and an increased concentration of IFN-γ and IL-17 within the lung tissue of infected mice. As seen in Figure 6, the γδ T cells that possibly produce IFN-γ are significantly increased on Day 5, and levels of IFN-γ production are still elevated through the end of the study on Day 7 (Figure 6A) with a similar trend observed with IL-17 producing γδ T cells (Figure 6B).

For the rest of the study, we proceeded to utilize the 10,000 CFU *F. tularensis* lethal dose as it was deemed sufficient to cause mouse morbidity without compromising the cellular architecture of the lung tissue, sample quality, or subsequent sample analysis.

### 3.2. Recruitment of Il-17 Producing γδ T Cells and Early Il-17 Production During Lvs Infec-Tion in the Lungs of Ift Immunized Mice

Studies have demonstrated that γδ T cells rapidly accumulate in the lungs following bacterial exposure and produce the cytokines IFN-γ and IL-17, which aid in bacterial clearance. Similar responses have been observed in mice, cattle, humans, chickens, and swine, highlighting γδ T cells’ role in early immune defense and pathogen clearance [18,19,20,21,22].

**Figure 7:**
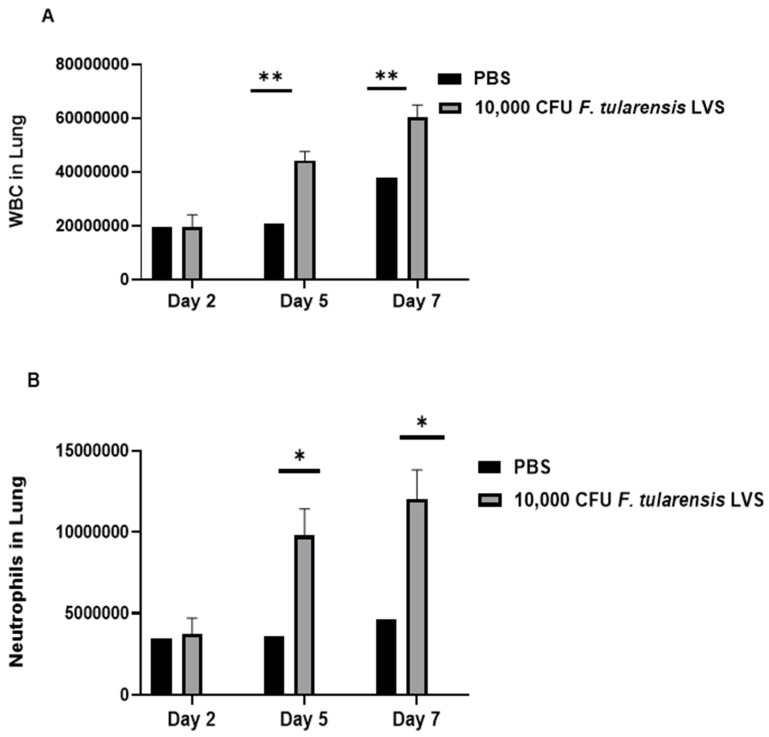
Primary infection with *F. tularensis* causes an increase in WBC and neutrophils. Inflammation due to intranasal dosing of 10,000 CFU of *F. tularensis* LVS caused WBC’s (**A**) including neutrophils (**B**) to increase significantly beginning on Day 5 post infection. Lungs were harvested and perfused from uninfected mice (PBS) as a baseline control. The WBC and neutrophil cell counts were obtained by analysis using an automated cell counter validated for use on mouse lung tissue. Three mice were used from each treatment group at each timepoint; the results are from one of the three experiments. (*) *p* < 0.1; (**) *p* < 0.05; bars represent SD.

Immune cell responses within the lung were assessed following intranasal *F. tularensis* LVS infection in naïve and i*Ft*-immunized mice. During primary LVS infection, a significant increase in total white blood cells (WBCs) was observed in the lung by Day 5 post-infection and remained elevated through Day 7 compared to PBS controls (Figure 7A). Neutrophil numbers were significantly increased over the same time frame (Figure 7B), with additional increases observed in monocytes, eosinophils, and basophils.

**Figure 8:**
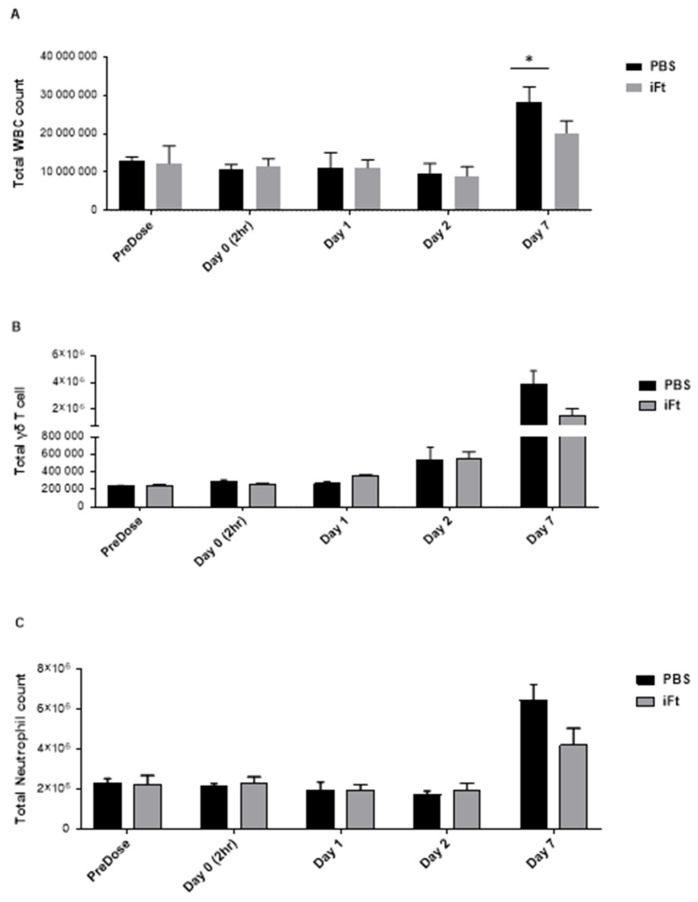
Immunized mice show decreases in WBC, γδ T cells, and neutrophils on Day 7. Mice immunized with i*Ft* has a decrease in WBCs (**A**), γδ T cells (**B**), and neutrophils (**C**) on Day 7 compared to the PBS group indicating less inflammation due to infection from *F. tularensis* LVS. Mice in the i*Ft* group were intranasally immunized with i*Ft* on Day 0, followed by a booster of the same immunization on Day 21. Challenge of 10,000 CFU *F. tularensis* LVS was then administered intranasally on Day 34 of the study. White blood cells and neutrophil counts were determined by an automated cell counter (Hemavet) validated for use with mouse tissue. Gamma-delta T cell counts were determined through flow cytometry and provided as an absolute count. Three mice were used for each group at each timepoint; the results are from one of the two experiments. (*) *p* < 0.1; bars represent SD.

To assess the effect of immunization, WBCs, γδ T cells, and neutrophils were evaluated in mice immunized intranasally with i*Ft* and subsequently challenged with lethal LVS. On Day 7 post-challenge, total WBCs, γδ T cells, and neutrophils were significantly increased in both immunized and control mice; however, all three populations were reduced in i*Ft*-immunized mice compared to PBS controls (Figure 8A–C, respectively). All i*Ft*-immunized mice survived with no physical signs of infection to the end of the study, whereas PBS control mice began showing signs of morbidity due to infection by Day 7.

**Figure 9:**
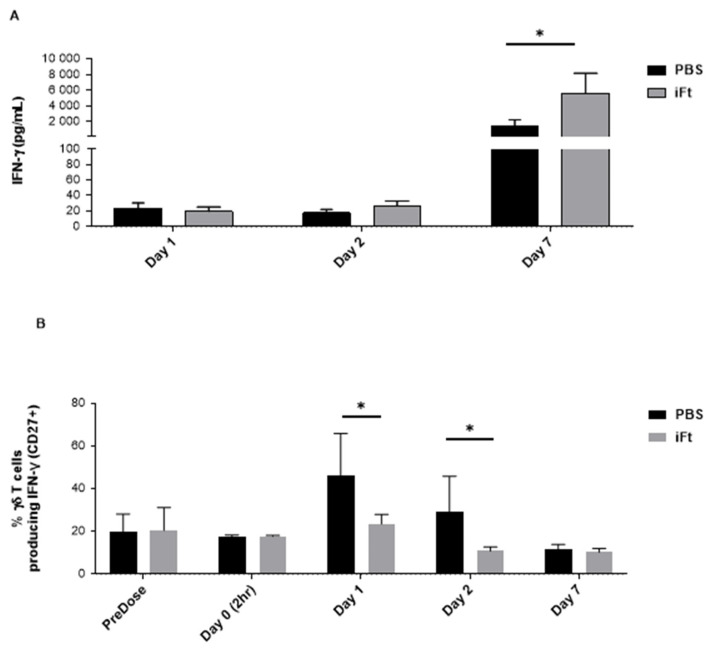
γδ T cells producing IFN-γ are not a primary source of IFN-γ in immunized mice. IFN-γ (**A**) was elevated in mice immunized with i*Ft* on Day 7 of the experiment. The concentration of IFN-γ in the lung was determined by ELISA. Cells positively labeled for γδ T and CD27 (**B**) in mice immunized with i*Ft* showed no correlation with IFN-γ. Mice were intranasally immunized with i*Ft* on Day 0, followed by a booster of i*Ft* on Day 21, challenge of 10,000 CFU of *F. tularensis* LVS was then administered intranasally on Day 34 of the study. Mice that were not immunized nor boosted but did receive the challenge administration of *F. tularensis* LVS are considered naïve (PBS) and serve as a baseline as to how infection progresses without immunization. Three mice were used for each group at each timepoint; the results are from one of the two experiments. Repeat analysis verified results. Analysis was determined by flow cytometry where γδ T cells (TCR-γδ GL-3) were labeled with CD27 (LG.3A10) for positive or negative cell receptor engagement. Cells TCR-γδ+CD27+ were analyzed as producing IFN-γ. (*) *p* < 0.1; bars represent SD.

IFN-γ levels in the lung were significantly increased on Day 7 post-challenge in i*Ft*-immunized mice (Figure 9A). Analysis of γδ T cells and CD27+ cells (possible IFN-γ producing γδ T cells) (Figure 9B) revealed that the IFN-γ response to infection post-immunization was not from γδ T cells.

**Figure 10:**
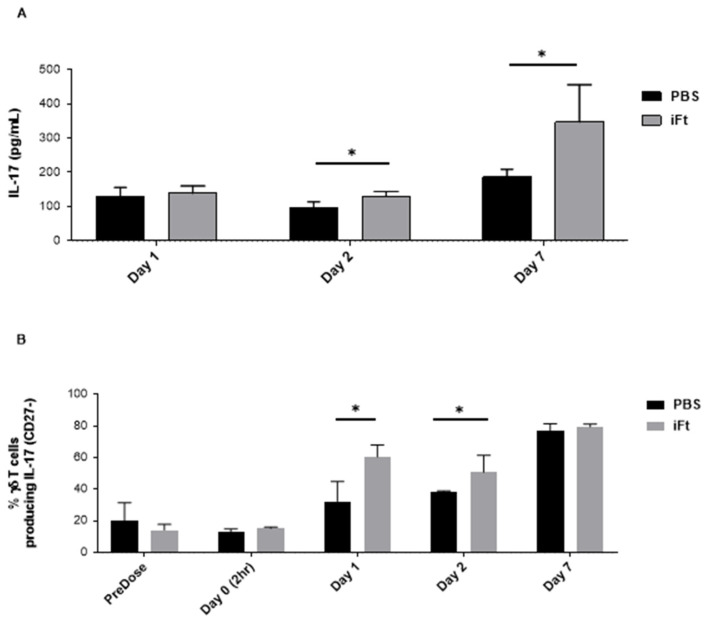
γδ T cells producing IL-17 increase in immunized mice. IL-17 concentration in mouse lungs is greater in mice immunized with i*Ft* on Day 7 showing a robust response to antigen challenge (**A**). The γδ T cells producing IL-17 also provide a robust response to antigen challenge as early as Day 1 and Day 2 (**B**). By Day 7 the γδ T cells producing IL-17 are the same as those mice not immunized (**B**). Mice in the i*Ft* group were intranasally immunized with i*Ft* on Day 0, followed by a booster of i*Ft* on Day 21. Challenge of 10,000 CFU *F. tularensis* LVS was then administered intranasally on Day 34 of the study (i*Ft* group). Three mice were used for each group at each timepoint; the results are from one of the two experiments. Repeat analysis verified results. IL-17 concentration in mouse lung tissue was obtained from ELISA analysis. Analysis was determined by flow cytometry where γδ T cells (TCR-γδ GL-3) were labeled with CD27 (LG.3A10) for positive or negative cell receptor engagement. Cells TCR-γδ+CD27− were analyzed as producing IL-17. (*) *p* < 0.1; bars represent SD.

However, early cytokine responses following LVS challenge revealed a significant increase in total pulmonary IL-17 in i*Ft*-immunized mice as early as Day 2 post-challenge compared to PBS controls (Figure 10A). This increase in IL-17 was also noted in a significant rise in CD27− γδ T cells (possibly IL-17 producing γδ T cells) on Day 1 post-challenge (Figure 10B). By Day 7, the percentage of CD27− γδ T cells was comparable between immunized and control groups, although total IL-17 levels remained significantly elevated in immunized mice.

This data shows that i*Ft* immunization with a decrease in WBCs, γδ T cells, and neutrophils provides protection following LVS challenge, accompanied by early increases in CD27− γδ T cells and sustained pulmonary IFN-γ and IL-17 levels.

### 3.3. Lung Il-17 Producing γδ T Cells Exhibit Both an Effector and Central Memory Phenotype in Ift-Immunized Mice

To assess whether intranasal immunization with i*Ft* induces γδ T cell memory responses that may contribute to protection against lethal LVS challenge, γδ T cell memory phenotypes were evaluated in the lung following infection. Since immunization with i*Ft* did not recruit significant numbers of CD27+ γδ T cells (possibly producing IFN-γ) in the lungs of immunized mice, memory cell analysis in this γδ T cell subpopulation was not conducted. However, i*Ft* immunized mice exhibited an early (Day 1) increase in IL-17 producing γδ T cells, coinciding with elevated lung IL-17 levels, suggesting a potential early role in protection due to memory formation.

γδ T cell memory populations were defined based on surface expression of CD44 and CD62L. Effector memory (EM) γδ T cells were identified as CD44+CD62L−, while central memory (CM) γδ T cells were identified as CD44+CD62L+. CD44 expression was used as a marker of antigen-experienced T cells, whereas CD62L expression was used to distinguish central memory populations. Additional characterization of IL-17 producing γδ T cells was performed based on CD27 expression.

**Figure 11:**
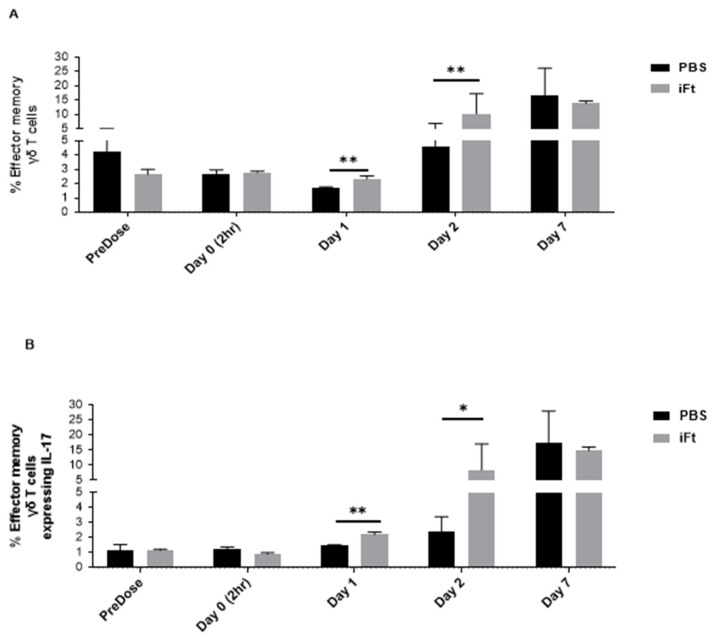
γδ effector memory T cells producing IL-17 (CD44+CD62L-TCRγδ+CD27−) increase in immunized mice. In the γδ effector memory T cells (**A**) there is a significant increase on Day 1 and a continued increase on Day 2 in mice immunized with i*Ft*. The γδ effector memory T cells in mice immunized with i*Ft*, shows a correlation with γδ T cells producing more IL-17 on both Day 1 and Day 2 (**B**). Mice were intranasally immunized with i*Ft* on Day 0, followed by a booster of i*Ft* on Day 21, challenge of 10,000 CFU of *F. tularensis* LVS was then administered intranasally on Day 34 of the study. Mice that were not immunized nor boosted but did receive the challenge administration of *F. tularensis* LVS are considered naïve (PBS) and serve as a baseline as to how infection progresses without immunization. Three mice were used for each group; the results are from one of the two experiments. Repeat analysis verified results. (*) *p* < 0.1; (**) *p* < 0.05; bars represent SD. Supplementary Figure S2 shows contour plot of Day 2, PBS compared to i*Ft* mice.

In Figure 11 we show a correlation between the γδ T cell effector memory population and the γδ T cell production of IL-17. As can be seen in Figure 11A, EM γδ T cells in mice immunized with i*Ft* are significantly elevated on Day 1 and 2 post challenge (Figure 11A). EM γδ T cells producing IL-17 (CD44+CD62L−TCRγδ+CD27−) (Figure 11B) are significantly increased on Day 1 through Day 2 in mice immunized with i*Ft,* correlating to the increase seen in the EM γδ T cells in Figure 11A and Supplementary Figure S2. These findings align with the increase in IL-17 production by γδ T cells observed on Day 1 post challenge seen in Figure 7, confirming that this early IL-17 response from γδ T cells likely originates from an effector memory γδ T cell population. There is no difference on Day 7, but the early reaction of γδ T cells producing IL-17 could be seen as possible protection in mice immunized with i*Ft*.

**Figure 12:**
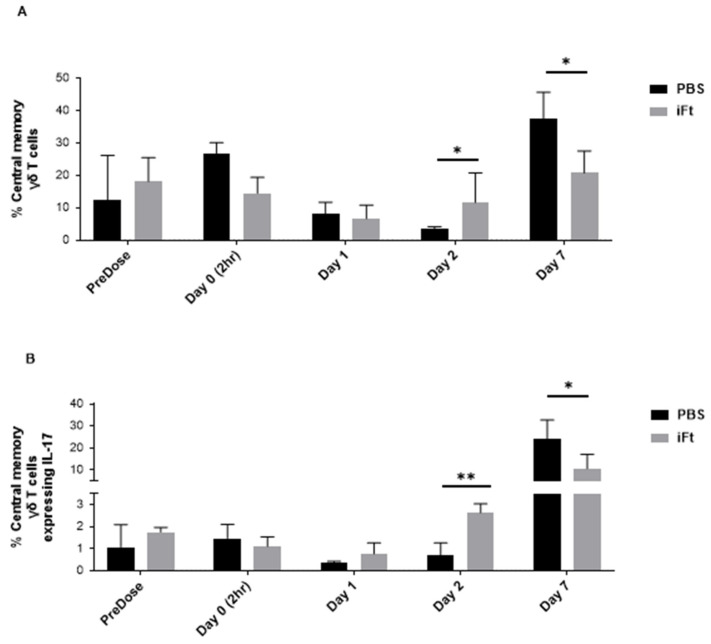
γδ central memory T cells producing IL-17 (CD44+CD62L+TCRγδ+CD27−) changes in immunized mice. In the γδ central memory T cells (**A**) there is a significant increase on Day 2 and a decrease on Day 7 in mice immunized with i*Ft*. The γδ central memory T cells in mice immunized with i*Ft*, shows a correlation with γδ T cells producing more IL-17 on both Day 2 and less on Day 7 (**B**). Mice were intranasally immunized with i*Ft* on Day 0, followed by a booster of i*Ft* on Day 21, challenge of 10,000 CFU of *F. tularensis* LVS was then administered intranasally on Day 34 of the study. Mice that were not immunized nor boosted but did receive the challenge administration of *F. tularensis* LVS are considered naïve (PBS) and serve as a baseline as to how infection progresses without immunization. Three mice were used for each group; the results are from one of the two experiments. Repeat analysis verified results. (*) *p* < 0.1; (**) *p* < 0.05; bars represent SD. Supplementary Figure S3 shows contour plot of Day 2, PBS compared to iFt mice.

Figure 12 compares the central memory (CM) γδ T cell population with IL-17 producing CM γδ T cells. As shown in Figure 12A, CM γδ T cells were significantly increased in i*Ft*-immunized mice by Day 2 post-challenge. Correspondingly, IL-17 production by CM γδ T cells (CD44+CD62L+TCRγδ+CD27−) (Figure 12B) was also significantly elevated at this time point, demonstrating a strong association between CM γδ T cell expansion and IL-17 production. By Day 7 post-challenge, CM γδ T cells remained elevated (Figure 12A); however, IL-17 production by these cells significantly decreased (Figure 12B and Supplementary Figure S3) in i*Ft*-immunized mice. Together, these findings suggest an early, coordinated increase in CM γδ T cells and IL-17 production following LVS challenge, followed by a decline in IL-17 output despite sustained CM γδ T cell levels.

Collectively, these data demonstrate that i*Ft* immunization induces both EM and CM γδ T cell populations in the lung that respond rapidly following LVS challenge. Effector memory γδ T cells producing IL-17 were elevated as early as Day 1 post-challenge, while IL-17 producing CM γδ T cells peaked by Day 2 post-challenge. The association between these γδ T cell memory subsets and IL-17 production supports a role for lung-resident γδ T cell memory responses in the early immune response to LVS infection in i*Ft*-immunized mice.

## 4. Discussion

The specific aim of this research was to investigate and characterize the immune response of γδ T cells upon intranasal immunization with i*Ft* followed by a LVS challenge. γδ T cells are known to bridge the innate and adaptive immune system’s response in a protective role against viruses, bacteria, wound healing, cancer therapy, and autoimmune disease in humans. A deeper understanding of how γδ T cells are recruited to LVS infection, both in primary infection and in response to immunization with i*Ft*, provides valuable insights into the role of this cell type.

Initial experiments showed that γδ T cells were rapidly and significantly recruited in the lungs of LVS-infected mice as early as Day 2 post-infection and remained elevated through Day 7. This response preceded the accumulation of conventional αβ T cells, which did not increase significantly until Day 7, suggesting that lung-resident γδ T cells may contribute to early containment of LVS. Analysis of cytokine responses revealed an increase in IFN-γ within lung tissue correlating with IFN-γ production by γδ T cells, with significant increases beginning on Day 5 through Day 7. Additionally, IL-17 levels in the lung increased beginning on Day 5 and remained elevated through the end of the study, closely correlating with the expansion of IL-17 producing γδ T cells.

The initial objective of the immunization study was to examine the relationship between γδ T cells producing IFN-γ or IL-17 and the recruitment of neutrophils, thereby assessing their potential contribution to early innate immune responses in the lungs of immunized mice. During primary LVS infection, a significant increase in total white blood cells and neutrophils was observed beginning on Day 5 post-infection, consistent with an inflammatory response. Following intranasal immunization with i*Ft* and subsequent LVS challenge, immunized mice exhibited reduced numbers of white blood cells, γδ T cells, and neutrophils in the lungs by Day 7 compared to control mice. This reduction likely reflects improved bacterial control and resolution of inflammation.

To determine whether early γδ T cell cytokine responses were associated with later reductions in inflammatory cells, we examined the recruitment of γδ T cells producing IFN-γ and IL-17 following challenge. Notably, IL-17 levels in the lungs of immunized mice were significantly elevated as early as Day 2 post-challenge compared to PBS controls. Further analysis demonstrated a significant increase in IL-17 producing γδ T cells on Day 1 post-challenge, indicating that γδ T cells contribute, at least in part, to this early IL-17 response. Given their localization at mucosal sites within the lung, these IL-17-producing γδ T cells are well positioned to respond rapidly to infection.

Contrary to our initial expectation, immunization did not elicit a marked increase in white blood cells, γδ T cells, or neutrophils before Day 7, suggesting that *F. tularensis* may initially evade immune detection following i*Ft* immunization or that protective immunity in this model relies on early cytokine signaling rather than overt cellular recruitment. It is also possible that subsequent activation of adaptive immune mechanisms, including CD4+ IFN-γ producing T cells, contributes to protection at later time points [4,42].

IFN-γ production in immunized mice was significantly increased at later stages of infection, particularly by Day 7 post-challenge. These findings are consistent with previous studies in our lab demonstrating that IFN-γ mediated protection following intranasal immunization targeting Fcγ receptors is largely driven by CD4+ effector T cells producing IFN-γ [4]. Thus, while IFN-γ remains essential for protection in our lab’s vaccine model, γδ T cells appear to play a limited role in IFN-γ production through i*Ft* immunization. Notably, γδ T cells exhibited minimal IFN-γ expression, indicating that γδ T cells preferentially adopt an IL-17 producing phenotype rather than an IFN-γ–producing one. This finding is consistent with findings from pulmonary infection models using *B. thailandensis*, further supporting a pivotal role for IL-17 producing γδ T cells in early mucosal immunity [16].

The contribution of IL-17 producing γδ T cells was most evident during the early phase of infection (Days 1–2), whereas by Day 7, IL-17 levels were comparable between groups, coinciding with the emergence of adaptive immune responses. While γδ T cells represent a key early source of IL-17, other innate-like T lymphocytes, including natural killer T (NKT) cells and mucosal-associated invariant T (MAIT) cells, may also contribute to this response [43]. Future studies employing IL-17 neutralization in i*Ft*-immunized mice, followed by LVS challenge, will be critical for defining the precise contribution of IL-17 and its cellular sources in protective immunity against *F. tularensis*.

Importantly, IL-17 levels remained significantly higher in immunized mice, possibly correlating with survival through the end of the study, supporting a protective role for IL-17 in this immunization model. However, IL-17 likely contributes to protection through multiple mechanisms beyond neutrophil recruitment alone. Previous work using the Fcγ receptor targeted vaccine model in our lab demonstrated reduced IL-10 production following immunization, a cytokine produced by *F. tularensis*-infected macrophages to evade immune detection, thereby permitting a more effective inflammatory response and bacterial clearance [3]. IL-10 is a critical regulator of IL-17, restraining neutrophil recruitment to limit lung pathology [44,45]. At the same time, IL-17 plays an essential role in promoting IL-12 driven Th1 immunity and inducing IFN-γ production, both of which are required for effective bacterial killing during LVS infection [45,46]. Together, these studies suggest that the reduced IL-10 environment induced by our current vaccine model may permit a transient, regulated IL-17 response that supports protection without progressing to pathological inflammation.

Further analysis in the immunization study provided evidence of a memory function in γδ T cells; memory being a key correlate of protection in vaccine development. Since we showed that γδ T cells are not the main producers of the IFN-γ observed in our immunization model, we focused on the memory function of IL-17-producing γδ T cells. γδ T cells producing IL-17 had EM as well as CM cell phenotypes. EM γδ T cells producing IL-17 were significantly elevated in immunized mice beginning on Day 1 and continued to be elevated on Day 2. By Day 7, the EM γδ T cells producing IL-17 in immunized mice were comparable to the control PBS mice. The CM γδ T cells producing IL-17 show a significant correlation to the CM γδ T cell response in immunized mice. By Day 7, there was a significant decrease in CM γδ T cells producing IL-17 in immunized mice, which correlated with reductions in CM γδ T cells and neutrophils. Collectively, these results demonstrate that i*Ft* immunization generates both EM and CM γδ T-cell memory subsets in the lung that respond rapidly to LVS challenge. The early and significant IL-17 production by EM γδ T cells on Day 1 and CM γδ T cells on Day 2 likely promotes timely bacterial clearance, providing a mechanistic basis for the enhanced protection observed in immunized mice. Thus, IL-17 producing lung-resident γδ T-cell memory populations appear to play a possible pivotal role in mediating immunity against LVS infection.

## 5. Conclusions

In this study, we provide a comprehensive in vivo characterization of γδ T cell responses following intranasal immunization with inactivated *F. tularensis* (i*Ft*) and subsequent lethal challenge with LVS. Our findings demonstrate enhanced recruitment of γδ T cells to the lung in immunized mice, accompanied by increased IL-17 production and the expansion of both central and effector memory γδ T cell populations. These results support a previously underappreciated role for γδ T cells in shaping protective mucosal immunity in pulmonary tularemia.

Mechanistically, our data suggest that γδ T cells contribute to early control of bacterial progression primarily through IL-17 mediated pathways. The elevated IL-17 response observed in immunized mice is consistent with enhanced recruitment and activation of neutrophils and other innate effector populations, which are critical for early bacterial containment in the lungs. Although γδ T cells were not the dominant producers of IFN-γ in this model, their rapid IL-17 driven activity highlights their importance in coordinating early inflammatory responses and bridging innate and adaptive immunity. These findings align with emerging evidence that lung-resident γδ T cells function as early sentinels that shape downstream immune responses during respiratory infection.

Importantly, we observed an increase in IL-17 producing γδ T cells exhibiting both effector and central memory phenotypes following i*Ft* immunization. This supports the concept that γδ T cells possess adaptive-like features and may contribute to durable protection upon pathogen re-exposure. The identification of memory-associated γδ T cell subsets in this model provides new insights into their potential role in vaccine-induced mucosal immunity and highlights their relevance as targets for next-generation vaccination strategies.

Collectively, this work advances the current understanding of γδ T cell function in pulmonary tularemia and identifies IL-17 producing γδ T cells as key contributors to the protective mechanisms elicited by intranasal immunization. These findings extend previous observations of γδ T cell involvement in early host defense and suggest that modulation of this population may enhance protective responses against intracellular respiratory pathogens.

Future studies will be required to define the precise mechanisms underlying γδ T cell mediated protection. Targeted depletion of γδ T cells and αβ T cells will be essential to delineate their relative contributions to vaccine-induced immunity. Further investigation of γδ T cell subsets, including Vδ1 and Vδ2 populations, will help distinguish tissue-resident from recruited cells and clarify their respective roles in pulmonary defense. In addition, in vivo neutralization of IL-17 will establish its functional importance in this model, while detailed characterization of neutrophil and macrophage activation will provide insight into downstream effector mechanisms. Finally, evaluating the balance between pro-inflammatory and regulatory pathways, including IL-17 and IL-10 signaling, may further reveal the immunological framework that governs protection following mucosal vaccination.

Together, these studies will refine our understanding of γδ T cell driven immunity and support the development of improved mucosal vaccines against *F. tularensis* and other respiratory pathogens.

## Data Availability

The raw data supporting the conclusions of this article will be made available by the authors upon request.

## Supplementary Materials

The following supporting information can be downloaded at https://www.mdpi.com/article/10.3390/vaccines14070590/s1: Figure S1: Contour plot analysis of memory γδ T cells; Figure S2: Contour plot of effector memory γδ T cells; Figure S3: Contour plot of central memory γδ T cells on Day 7.
