# Supplementary material for: Potential Roles of Gamma-Delta T Cells in a Bacterial Immun-Ization Model
Source: Vaccines (Basel). 2026 Jul 1;14(7):590. doi: 10.3390/vaccines14070590 (PMC13418333; doi:10.3390/vaccines14070590)
Supplement: Supplementary file 1 [file vaccines-14-00590-s001.zip › vaccines-4370987-supplementary.pdf]

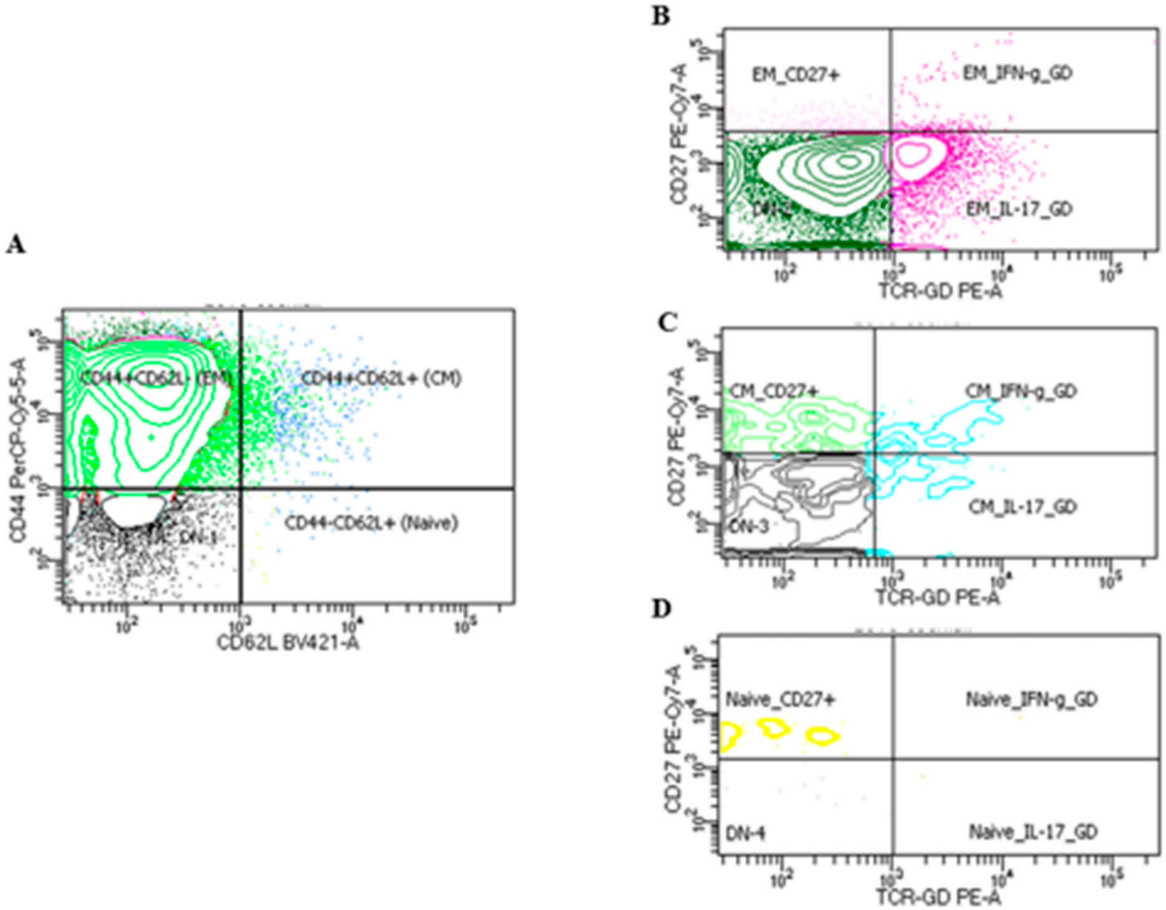

Supplementary Figure S1 – Contour plot analysis of memory γδ T cells – Contour analysis consisted with labeling cells with CD44 and CD62 for determination of effector memory cells (EM) or central memory cells (CM) (A). The EM, CM, and Naïve quadrants were then labeled with CD27 and TCR γδ (B, C, and D respectively). If the cells stained positively for CD27, the literature indicates those cells produce IFN-γ. If the cells do not stain for CD27, the literature indicates those cells produce IL-17 [38].

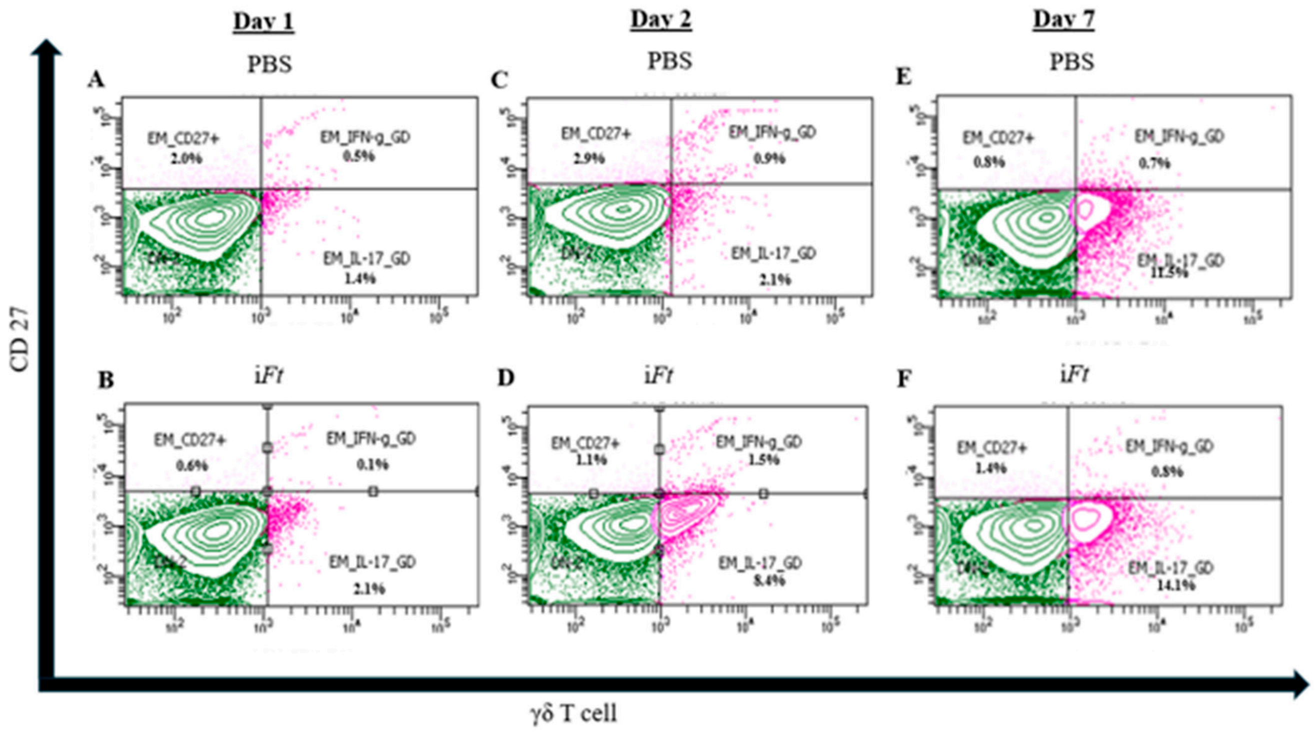

Supplementary Figure S2 – Contour plot of effector memory γδ T cells. Antigen specific changes in the γδ effector memory T cells and the production of IL-17 (CD44+CD62L-TCRγδ+CD27-). The γδ effector memory T cells in mice immunized with i*Ft* (B, D, F), was significantly increased over the PBS mice (A, C, E) on Day 1 and Day 2. Mice were intranasally immunized with i*Ft* on Day 0, followed by a booster of i*Ft* on Day 21, challenge of 10,000 CFU of *F. tularensis* LVS was then administered intranasally on Day 34 of the study. Mice that were not immunized nor boosted but did receive the challenge administration of *F. tularensis* LVS are considered naïve (PBS) and serve as a baseline as to how infection progresses without immunization. Current flow cytometry capture is one of three mice used in each group; the results are from one of the two experiments. Repeat analysis verified results.

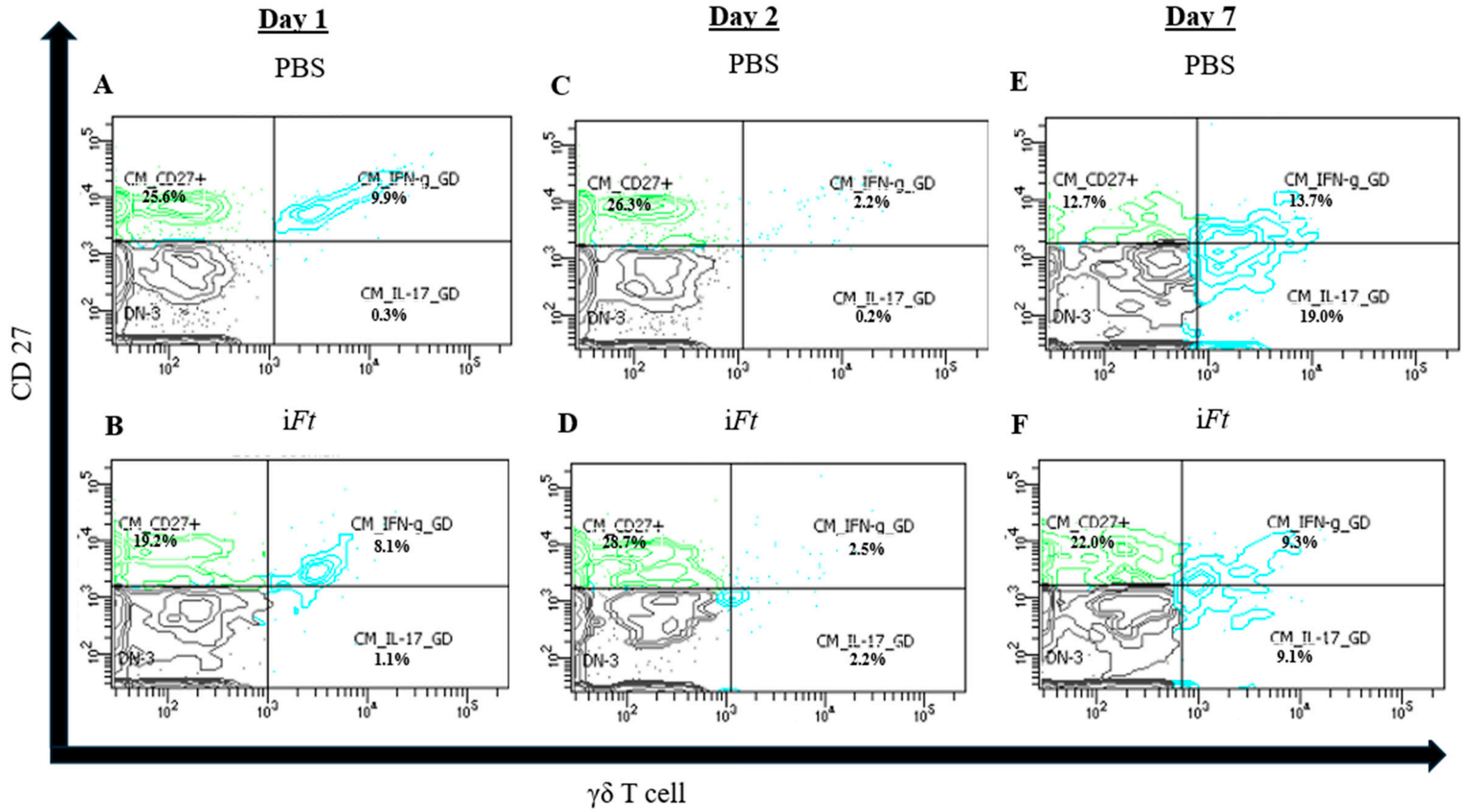

Supplementary Figure S3 – Contour plot of central memory γδ T cells on Day 7. Antigen specific changes in the γδ central memory T cells and the production of IL-17 (CD44+CD62L+TCRγδ+CD27-). The γδ central memory T cells in mice immunized with i*Ft* (B, D, F), compared to the PBS mice (A, C, E) were significantly increased on Day 2 and significantly decreased on Day 7. Mice were intranasally immunized with i*Ft* on Day 0, followed by a booster of i*Ft* on Day 21, challenge of 10,000 CFU of *F. tularensis* LVS was then administered intranasally on Day 34 of the study. Mice that were not immunized nor boosted but did receive the challenge administration of *F. tularensis* LVS are considered naïve (PBS) and serve as a baseline as to how infection progresses without immunization. Current flow cytometry capture is one of three mice used in each group; the results are from one of the two experiments. Repeat analysis verified results.
